# Cytomegalovirus-Infected Cells Resist T Cell Mediated Killing in an HLA-Recognition Independent Manner

**DOI:** 10.3389/fmicb.2016.00844

**Published:** 2016-06-09

**Authors:** Julia Proff, Christian Walterskirchen, Charlotte Brey, Rene Geyeregger, Florian Full, Armin Ensser, Manfred Lehner, Wolfgang Holter

**Affiliations:** ^1^Children’s Cancer Research Institute, St. Anna KinderkrebsforschungVienna, Austria; ^2^Children’s University Hospital, Universitätsklinikum ErlangenErlangen, Germany; ^3^Institute for Clinical and Molecular Virology, Universitätsklinikum ErlangenErlangen, Germany; ^4^Department of Microbiology, The University of ChicagoChicago, IL, USA; ^5^Department of Pediatrics, St. Anna Kinderspital, Medical University of ViennaVienna, Austria

**Keywords:** herpesvirus, cytomegalovirus, immune evasion, chimeric antigen receptor, anti-apoptotic, UL36, UL37x1, glycoprotein B

## Abstract

In order to explore the potential of HLA-independent T cell therapy for human cytomegalovirus (HCMV) infections, we developed a chimeric antigen receptor (CAR) directed against the HCMV encoded glycoprotein B (gB), which is expressed at high levels on the surface of infected cells. T cells engineered with this anti-gB CAR recognized HCMV-infected cells and released cytokines and cytotoxic granules. Unexpectedly, and in contrast to analogous approaches for HIV, Hepatitis B or Hepatitis C virus, we found that HCMV-infected cells were resistant to killing by the CAR-modified T cells. In order to elucidate whether this phenomenon was restricted to the use of CARs, we extended our experiments to T cell receptor (TCR)-mediated recognition of infected cells. To this end we infected fibroblasts with HCMV-strains deficient in viral inhibitors of antigenic peptide presentation and targeted these HLA-class I expressing peptide-loaded infected cells with peptide-specific cytotoxic T cells (CTLs). Despite strong degranulation and cytokine production by the T cells, we again found significant inhibition of lysis of HCMV-infected cells. Impairment of cell lysis became detectable 1 day after HCMV infection and gradually increased during the following 3 days. We thus postulate that viral anti-apoptotic factors, known to inhibit suicide of infected host cells, have evolved additional functions to directly abrogate T cell cytotoxicity. In line with this hypothesis, CAR-T cell cytotoxicity was strongly inhibited in non-infected fibroblasts by expression of the HCMV-protein UL37x1, and even more so by additional expression of UL36. Our data extend the current knowledge on Betaherpesviral evasion from T cell immunity and show for the first time that, beyond impaired antigen presentation, infected cells are efficiently protected by direct blockade of cytotoxic effector functions through viral proteins.

## Introduction

Human cytomegalovirus infection after hematopoietic stem cell transplantation is associated with substantial morbidity and mortality. Antiviral chemotherapy is prophylactically and preemptively used early after transplantation but frequently results in toxicity and selection of drug resistant viruses. Moreover, it does not prevent all complications associated with HCMV (Boeckh and Ljungman, 2009; Boutolleau et al., 2009). Adoptive transfer of HCMV-specific memory T cells has been successfully applied, however, generation of specific T cells from seronegative donors was accomplished only recently (Hanley et al., 2015; Nicholson and Peggs, 2015). We hypothesized that HCMV could be targeted in an HLA-independent manner by CAR-T cells directed against intact viral proteins, such as gB, that are displayed on the surface of HCMV-infected cells. This strategy would circumvent the time-consuming enrichment and expansion of preexisting HCMV-specific memory T cells, and it would ease the application of adoptive T cell therapy in the high-risk constellation of an HCMV seronegative donor and an HCMV seropositive transplant patient.

The CAR approach has been applied early for treatment of HIV, where some efficacy has been shown even in patients (Mitsuyasu et al., 2000). Recently, the efficacy of CAR-expressing T cells to eliminate virus-infected cells has also been demonstrated for Hepatitis B and Hepatitis C (Krebs et al., 2013; Sautto et al., 2016). We have previously constructed a functional CAR targeting the conserved AD1 region of gB, which mediated T cell activation in response to HCMV-infected cells (Full et al., 2010).

Interestingly, systematic investigation of the cytotoxic potential of these cells showed that T cells equipped with this CAR could not directly eliminate cells infected with this virus. This was unexpected, since our approach was designed to circumvent the known Betaherpesvirus evasion of T cell killing by reduced MHC class I antigen-presentation (Crough and Khanna, 2009; Halenius et al., 2015).

Our study with CAR-T cells now explicitly shows that a virus can evade T cell immunity by blockade of cytotoxic effector functions and that this can be directly mediated by a viral protein. To our knowledge this is the first evidence of such a mechanism, which likely is not confined to HCMV but could be more common in viral immune evasion.

## Materials and Methods

### Cells and Cell Lines

Purification of PBMCs from leukapheresis obtained from voluntary healthy donors was undertaken by density gradient centrifugation using Ficoll. T cells were isolated from PBMCs by negative selection of CD3^pos^ cells using the Dynabeads Untouched Human^TM^ T cell Kit (Life Technologies) according to the manufacturer’s instructions. HFF were isolated from foreskin of circumcised donors after mechanic disruption by enzymatic digestion with 5 mg/ml Collagenase D, 25 U/ml Dispase, and 0.05% Trypsin/EDTA and were cultured in R-10 medium, consisting of RPMI GlutaMAX^TM^ (Life Technologies) supplemented with 10% FBS, 100 U/ml penicillin and 100 μg/ml streptomycin. Isolated HFF were used for experiments until passage 20. Written informed consent was obtained from every voluntary donor and each study protocol was approved by the local ethics committee (Friedrich-Alexander-Universität Erlangen-Nürnberg no. 2247, Medizinische Universität Wien no. 514/2011). Phoenix and 293T cells were cultured in DMEM (Life Technologies) supplemented with 10% FCS and 100 U/ml penicillin and 100 μg/ml streptomycin, and the hybridoma cell line “gB 27-287” (a kind gift from M. Mach, Institute of Virology, Universitätsklinikum Erlangen, Germany) was kept in R-10.

### Activation and Expansion of T Cells

T cells were stimulated with either Human T-Activator CD3/CD28 Dynabeads (Life Technologies) according to the manufacturer’s instructions or with peptide-loaded matured Dendritic cells (DC). Briefly, monocytes were enriched by adherence in RPMI GlutaMAX^TM^ supplemented with 2% human serum for 2 h and cultivated afterward in R-10 supplemented with 500 U/ml IL-4 and 500 U/ml IL-4 GM-CSF (both from Peprotech). On day 5, DCs were maturated by the addition of 250 U/ml IL-4, 250 U/ml IL-4 GM-CSF, 10 ng/ml IL-1β (R&D Systems), 10 ng/ml TNF (Miltenyi), 1000 U/ml IL-6 (Peprotech), and 1 μg/ml Prostaglandin E_2_ (Sigma). Mature DCs were loaded on day 7 with 5 μg/ml of the HLA-A^∗^0201 restricted EBV-BMLF1 peptide GLCTLVAML (Genscript). Peptide-pulsed DCs were mixed with T cells at a ratio of 1:4 for stimulation of EBV-specific T cells and seeded at a density of 0.5 × 10^6^ T cells/ml RPMI GlutaMAX^TM^ supplemented with 10% human serum, 100 U/ml IL-2 (Peprotech), 50 μM β-mercaptoethanol, 100 U/ml penicillin and 100 μg/ml streptomycin, 1 mM sodium pyruvate, 1x vitamins and 1x non-essential amino acids (Gibco). Stimulation of T cells with mature peptide-pulsed DCs was performed weekly. After the second stimulation CD8^pos^ T cells were positively selected using Dynabeads FlowComp Human CD8 Kit (Life Technologies) according to the manufacturer’s protocol and were cultured after the third antigen-specific stimulation in medium supplemented with 10% FCS instead of 10% HS. CD3/CD28-activated T cells were kept in R-10 supplemented with 100 U/ml IL-2, 50 μM β-mercaptoethanol, 100 U/ml penicillin and 100 μg/ml streptomycin, 1 mM sodium pyruvate, 1x vitamins and 1x non-essential amino acid. EBV-specific as well as CD3/CD28-activated T cells were used between days 25 and 32 for experiments.

### Viruses

Human cytomegalovirus strain AD169 encoding GFP was provided by M. Marschall (Universitätsklinikum Erlangen, Erlangen, Germany; Marschall et al., 2000), GFP-recombinant strains AD169 ΔUS2-11 (Falk et al., 2002) and Towne ΔUS1-12 (Marchini et al., 2001) were kind gifts from B. Plachter (Universitätsmedizin, Johannes Gutenberg-Universität Mainz, Mainz, Germany) and H. Zhu (Rutgers New Jersey Medical School, Newark, USA), respectively. Infectious HCMV supernatants were obtained by infection of semi-confluent HFF with an MOI 0.1 and collected after 11–14 days, centrifuged, and stored at -80°C until further use. Titration of HCMV stocks was performed by using the limiting dilution method according to Reed and Munch. In the experiments, confluent HFF were infected with MOI 5 unless otherwise specified and used at the indicated time points after infection.

### Flow Cytometric Analysis

Flow cytometric analysis of the T cell phenotype was performed using the following mAbs: CD3 (clone SK7), CD4 (clone OKT4,), CD8 (clone RPA-T8), CD27 (clone M-T271), CD28 (clone CD28.2), CD56 (clone NCAM1.2), CD62L (clone DREG-56). CAR-expression in T cells was detected with a biotinylated anti-human IgG mab (clone JDC-10) continued by incubation with PE-conjugated streptavidin. Cell surface expression of HCMV-gB in viable HFF (propidium iodide^neg^ or 7-AAD^neg^) was detected by incubation with supernatants from hybridoma gB 27-287 and a PE- or APC-conjugated anti-mouse antibody. HLA-A^∗^02 surface expression in viable HFF was determined by using an APC-labeled HLA-A^∗^02 antibody (clone BB7.2). Non-infected and HCMV-infected HFF were blocked with human serum from a HCMV-seronegative individual before staining with antibodies. Intracellular expression of perforin and granzymes in T cells or UL37x1 in HFF, respectively, was analyzed after fixation with formaldehyde and permeabilization with saponin followed by incubation with monoclonal antibodies directed against perforin (clone dG9), granzyme A (clone CB9), granzyme B (clone GB11), granzyme K (clone G3H69), granzyme M (clone 4B2G4), and the myc-Tag (clone Sc-40), respectively. Cells were counted using Accu chek counting beads (Life Technologies), whereby propidium iodide or Annexin V-FITC was added for exclusion of dead cells. BD LSR Fortessa was used for measurements and BD FacsDiva and FlowJo were used for data analysis.

### *In Vitro* Transcription and Electroporation of mRNA

DNA templates for *in vitro* transcription of mRNA were generated by linearization of plasmids pGEM4Z encoding the CARs directed against HCMV-gB, CEA, and chNKG2D (Full et al., 2010; Lehner et al., 2012). The mRNA encoding for UL36 was generated from a PCR product amplified from pLV-EF1α-MCS-UL36-IRES-puro kindly provided by E. Mocarski (Emory University School of Medicine, Atlanta, USA; McCormick et al., 2010) using two specific primer pairs for amplification of UL36 exons (gcttacgtctgctgtcaggag, cgtgaggaatttcgacatttaatacgactcactatagggttccatttcaggtgtcgtgacgataccgtcgagattaattaaatttcagttgttcatgtaaacgtgtg, tcctgacagcagacgtaagcaccttgcgaacagacggtg) followed by Gibson assembly (NEB). The fusion construct of the gB-ectodomain and EpCAM transmembrane/cytoplasmic proportion was transcribed from a purified PCR product, which was amplified from a bacmid encoding a respective recombinant murine cytomegalovirus. *In vitro* mRNA transcription was performed with the mMessage mMachine T7 Ultra Kit (Ambion) according to the manufacturer’s instructions followed by RNA purification with the RNeasy Kit (Qiagen). For electroporation of the mRNA, T cells and 293T cells were resuspended in phenol-free Opti-MEM at a density of 8 × 10^7^/ml (T cells) or 0.5 × 10^7^/ml (293T cells). HFF were detached with Trypsin/EDTA and resuspended at a density of 0.5 × 10^6^/400 μl in GenePulser^®^ Electroporation Buffer Reagent (Bio-Rad). Electroporation was performed with 100 μl cell suspension (T cells and 293T cells) or 400 μl cell suspension (HFF) in a 4 mm electroporation cuvette after addition of 10 μg mRNA using the GenePulser square wave protocol (Gene Pulser Bio-Rad, conditions: 500 V and 5 ms for T cells and HFF, 500 V and 3 ms for 293T cells).

### Transduction of HFF with Viral Vectors

Lenti- or retroviral particles containing supernatants were produced by transfection of 293T or Phoenix cells, respectively. The lentiviral vector pWPI encoding EpCAM-gB was co-transfected with psPAX (Addgene 12260) and MD2.G (Addgene 12259) in a ratio of 4:3:1 in 293T cells using 3.5 μg/ml PEI. Phoenix cells were transfected with the retroviral vector pLNCX UL37x1 or pLNCX GFP [kindly provided by McCormick et al. (2005)] and pMD2.G (4:1 ratio) by addition of 3.5 μg/ml PEI. Viral supernatants were harvested after 48 h, cleared of cell debris, concentrated using Spin-X^®^ UF Concentrator (100,000 Dalton; Corning), aliquoted and stored at -80°C until further use. HFF were spinoculated three times with retroviral supernatants diluted 1:1 with fresh media (1500 × *g*, 33°C, 4 h). Transduced cells were either selected by addition of 4 μg/ml Geneticin (pLNCX-vector) or by flow cytometric cell sorting using BD FACSAria, respectively.

### Cytotoxicity Assays

The lytic potential of T cells was analyzed in a non-radioactive Eu-release assay (Blomberg et al., 1996) or by determination of apoptotic target cells using Annexin V by flow cytometry. Eu release assay: target cells were detached with EDTA and labeled with Eu as previously described (Lehner et al., 2007). 2500 Eu-labeled target cells were co-incubated with effector T cells at the indicated effector:target(E:T)-ratio for 4 h at 37°C. Eu-release by target cells into supernatant was analyzed by addition of 200 μl Enhancement Solution (Perkin Elmer) to 25 μl co-culture supernatant using time-resolved fluorometry (TRF; Victor, Wallac). Specific target cell lysis was calculated using the following formula: specific lysis [%] = (Eu release in co-culture – spontaneous Eu release)/(maximum Eu release – spontaneous Eu release) ^∗^100. Flow cytometric apoptosis assay: Target cells were seeded at a density of 10,000 target cells/96-well after detachment. T cells were labeled with 10 μM SNARF-1^®^ (carboxylic acid acetate succinyl ester; Life Technologies) in PBS for 15 min and added to the target cells at the indicated E:T-ratios. After 4 or 20 h of co-culture at 37 °C target cells were harvested with Trypsin/EDTA, washed once with PBS 0.2% HA, 0.02% Na-Azide and resuspended in 1x Annexin binding buffer (eBioscience). Cells were analyzed after addition of 5 μl Annexin V and 5 μl 7-AAD in a flow cytometric analysis. Apoptotic cells were determined by exclusion of T cells and gating on Annexin V^pos^ target cells. Only GFP^pos^ cells were considered in the case of HCMV-infected HFF. Apoptosis in UL37x1-transduced HFF fraction was calculated after flow cytometric analysis of intracellular UL37x1-myc expression in HFF (60.4–94.8%) using the formula: Annexin V^pos^ cells [%] = 100/myc^pos^ HFF [%] ^∗^ (Annexin V^pos^ UL37x1-HFF detected by FACS [%]–X); X = (100 – myc^pos^ [%]) ^∗^ Annexin V^pos^ mock-HFF detected by FACS [%]/100.

### Degranulation Assay

Flow cytometric analysis of CD8^pos^ T cell degranulation was performed by extracellular staining of CD107a as previously described (Betts et al., 2003). Briefly, 50,000 T cells were co-incubated with 50,000 target cells in the presence of PE-labeled CD107a in RPMI supplemented with 10% FCS and 10% HS. Monensin was added to a final concentration of 5 μM after 1 h of co-incubation at 37°C. After 4 h, the T cells were harvested and additionally stained with antibodies directed against CD3, CD4 and CD8.

### ELISA

After co-culturing 60,000 T cells with 30,000 target cells in 200 μl RPMI1640 for 4 h, the amounts of secreted IFN-γ and TNF were determined using ELISA kits from eBioscience and Mabtech, respectively.

### Statistical Analysis

Statistical significance was calculated using the paired two-tailed Student’s *t*-test (^∗∗∗^ = *p* < 0.001, ^∗∗^ = *p* < 0.01, ^∗^ = *p* < 0.05).

## Results

### CAR-T Cells Directed against HCMV-gB Do Not Lyse HCMV-Infected Cells

We previously demonstrated that our HCMV-gB specific CAR is capable of mediating efficient lysis of gB transfected 293T cells and of inducing cytokine release from the CAR-T cells in response to HCMV-infected HFF (Full et al., 2010). In previous unpublished experiments with anti-CD3 activated T cells, however, we observed weak or absent lytic activity of CAR-T cells against HCMV-infected HFF 3 days after infection (data not shown), although gB was strongly expressed on the surface of infected cells at that time point (Supplementary Figure S1A).

To investigate whether the observed absence of lysis of infected cells expressing the target antigen was due to low T cell efficacy or a peculiarity of HCMV infection, we decided to thoroughly investigate this issue by conducting experiments with effector T cells activated (A) in an antigen-independent manner by αCD3/αCD28 antibody-coated beads, or (B) enriched from a preexisting memory pool of Epstein-Barr virus (EBV)-specific effector T cells. For the latter we employed a previously established culture system relying on weekly restimulation with peptide-loaded DC, which resulted in efficient expansion of antigen-specific cytotoxic memory T lymphocytes (CTLs; routinely above 50%; Supplementary Figure S1B; Lehner et al., 2007). The cultures were depleted of NK cells, and if necessary sorted for CD8^pos^ T cells, finally resulting effector cell populations consisting of 56.3–98.8% CD8^pos^ T cells (αCD3/αCD28-activated T cells) or 92.2–98.2% CD8^pos^ T cells (EBV-specific CTLs, not shown), and < 1.0% NK cells. EBV-specific effectors were additionally chosen because of a more advanced differentiation stage (loss of CD62L and CD45RA; upregulation of CD45RO; Supplementary Figures S1C,D). A detailed analysis of the expression profile of the cytotoxic effector molecules perforin and granzymes A, B, K, and M, however, showed a pattern which was very similar to that of αCD3/αCD28-activated T cells (Supplementary Figure S1E).

**FIGURE 1:**
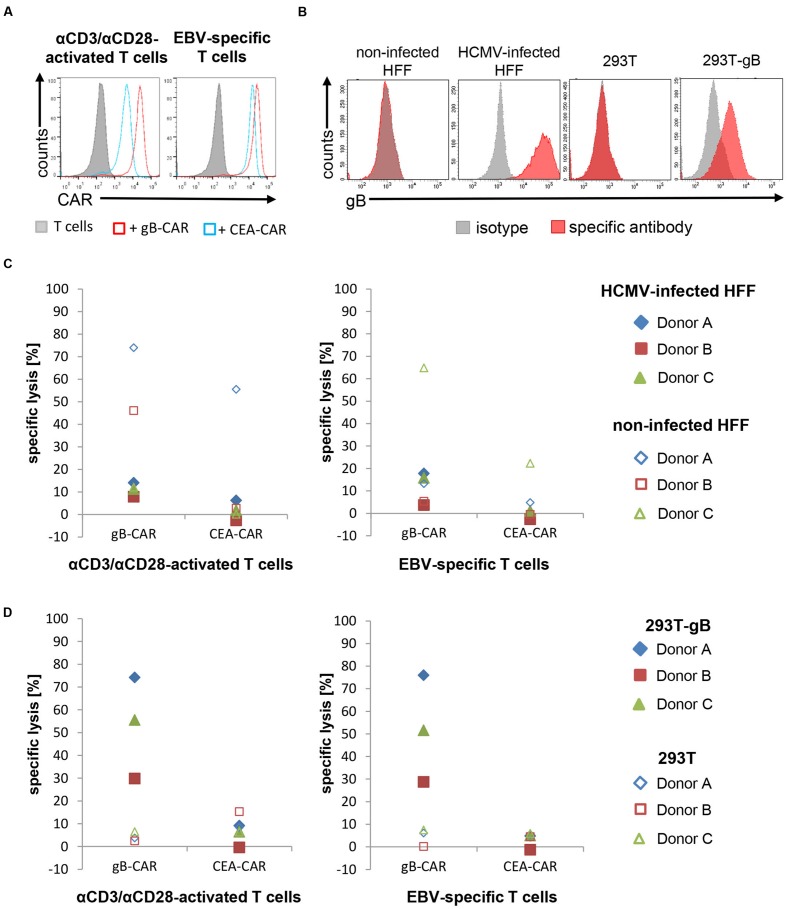
**Lysis of HCMV-infected HFF by CAR-T cells is strongly reduced. (A)** The histograms shows the expression of the different CARs in αCD3/αCD28-activated or EBV-specific T cells, respectively, 20 h after mRNA electroporation of a representative donor (EBV-specific T cells *n* = 6; 4 donors; αCD3/αCD28-activated *n* = 3; 3 donors). **(B)** Flow cytometric analysis of the gB-expression in 293T cells, gB-expressing 293T (293T-gB) cells, non-infected and HCMV-infected HFF. Shown is a representative histogram of one experiment (*n* = 3). **(C,D)** The diagrams show the lytic potential of redirected αCD3/αCD28-activated or EBV-specific T cells targeting HCMV-infected HFF (C) or gB-expressing 293T (293T-gB; **(D)** analyzed in a 4 h Eu release assay (E:T-ratio referring to CD8^pos^ T cells = 25:1). 293T cells, non-infected HFF were used as target cell controls, CEA-CAR-expressing EBV-specific T cells were used as controls for CAR-T cells. **(B–D)** HCMV-infected HFF in all shown experiments were used at 4 days after infection (AD169, MOI 5).

The polyclonally activated and EBV-specific T cells were transiently transfected with either the gB-specific CAR or an irrelevant CAR specific for the CEA by electroporation of CAR-encoding mRNA (CAR expression shown in **Figure 1A**; Hombach et al., 2001). Lytic activity of these CAR-T cells was determined 1 day after electroporation using HCMV-infected HFF and gB-expressing 293T as target cells (gB-expression is shown in **Figure 1B**). Unexpectedly, neither type of effector T cells achieved substantial lysis of HFF 4 days after infection (**Figure 1C**), not even with an effector:target(E:T)-ratio of 25:1. However, the same effectors strongly lysed gB-expressing 293T cells, although gB-expression in these target cells was lower than on infected HFFs (**Figure 1D**). Notably, non-specific killing, which was frequently observed with non-infected fibroblasts, also seemed to be inhibited by HCMV, since it never occurred in infected fibroblasts (**Figure 1C**).

**FIGURE 2:**
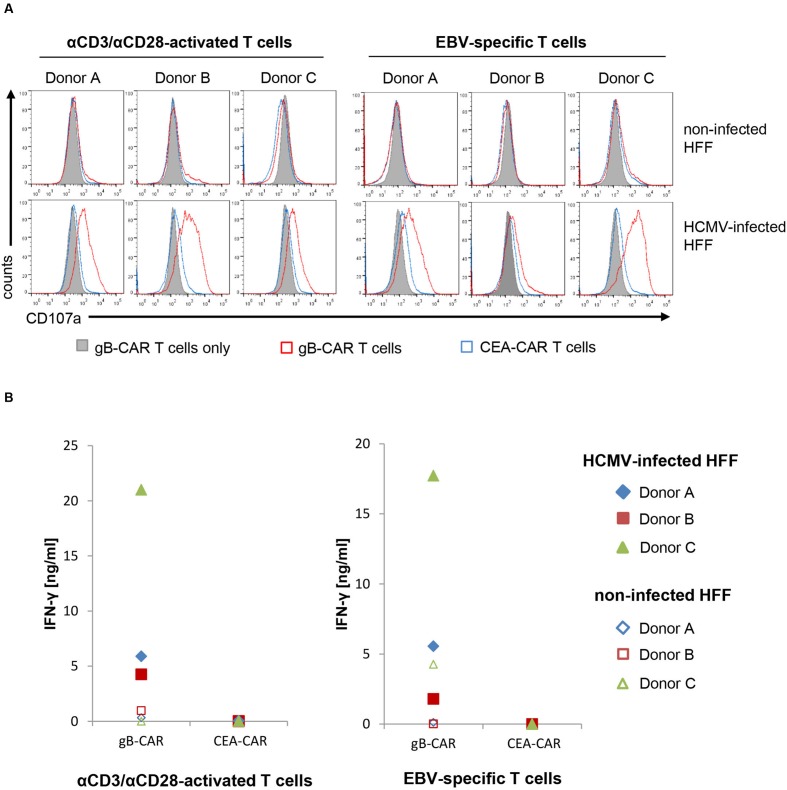
**CAR-T cells are activated by HCMV-infected HFF. (A)** The histograms show the degranulation of CD8^+^ CAR-expressing αCD3/αCD28-activated or EBV-specific T cells, respectively, after co-incubation with non-infected and HCMV-infected HFF, as analyzed by flow cytometric detection of CD107a cell surface expression. **(B)** Levels of IFN-γ secreted into the supernatants of co-cultures of CAR-modified T cells and non-infected or HCMV-infected, respectively. **(A,B)** HCMV-infected HFF in all shown experiments were used 4 days after infection (AD169, MOI 5).

We further determined the level of T cell degranulation and cytokine production in order to exclude that the observed lack of lysis of HCMV-infected HFF is due to inhibition of T cell activation. **Figure 2A** illustrates that the gB-specific CAR triggers degranulation in both effector populations in co-culture with infected HFF, whereas the T cells did not degranulate in the control conditions. Similarly, IFN-γ production was triggered by the gB-specific CAR in response to infected HFF, whereas there was no or low-level production under control conditions (**Figure 2B**); analysis of TNF showed the same pattern (data not shown). Notably, the readout of these effector functions was congruent between the αCD3/αCD28-activated T cells and the EBV-specific CTLs, and showed a high specificity for gB-recognition, in contrast to cytotoxicity with frequently also unspecific lysis of non-infected HFF (**Figure 1C**).

Together, these data clearly show that both polyclonally expanded as well as antigen-specific memory T cells, when engrafted with a gB-specific CAR, cannot lyse infected HFF despite detectable CAR-mediated specific activation of the T cells.

### HCMV also Blocks TCR-Mediated Lysis Despite Activation of T Cells

In the next step we wanted to exclude that a possible defective function of our gB-specific CAR was responsible for the observed lack of lysis. For this reason we again employed EBV BMLF1-peptide-specific CTLs, which this time, however, were directed via their TCR to HLA-A^∗^0201 matched fibroblasts loaded with a saturating concentration of the same BMLF1-peptide that was used for T cell enrichment. In addition, we used a recombinant variant of the HCMV-strain Ad169 (Ad169ΔUS2-11), which contained extended deletions in the US-gene region encoding the known viral inhibitors of antigenic peptide presentation, in order to prevent down-regulation of MHC class I molecules during infection (Supplementary Figure S2A; Jones et al., 1995; Besold et al., 2009).

**FIGURE 3:**
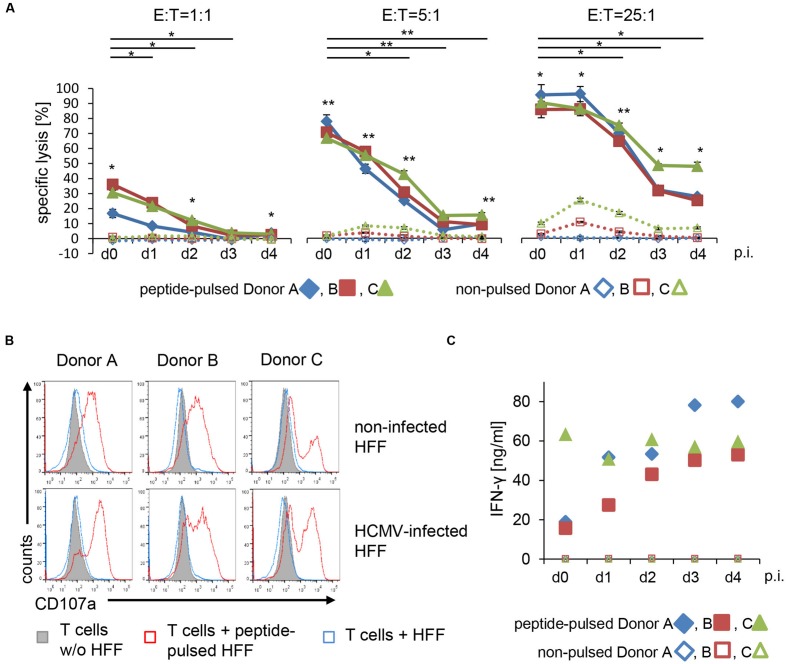
**T cell receptor-mediated lysis of HFF is reduced by HCMV starting from 1 day after infection. (A)** TCR-mediated lysis of non-infected and HCMV-infected HFF (AD169 ΔUS2-11, MOI 5) by EBV-specific T cells (E:T = 1:1; 5:1; 25:1) was analyzed in a 4 h Eu release assay at the indicated time points after infection (p.i. = after infection; three technical replicates with each donor). Target cells were pulsed with BMLF1-peptide (2 μM GLCTLVAML) for 2 h before co-incubation with EBV-specific T cells or left untreated. **(B)** The histograms show the degranulation of EBV-specific T cells after co-incubation with either untreated or peptide-pulsed non-infected and HCMV-infected HFF (AD169 ΔUS2-11, MOI 5, d4 p.i.), as analyzed by flow cytometric detection of CD107a at the cell surface. **(C)** Levels of IFN-γ secreted into the supernatants of co-cultures of EBV-specific T cells and peptide-pulsed or non-pulsed HFF, respectively (non-infected or 1–4 days after infection with AD169 ΔUS2-11, MOI 5, as indicated).

In this CAR-independent experimental setup HCMV-infection again resulted in a striking resistance of the HFF to lysis by the T cells (**Figure 3A**). Lysis-inhibition of the peptide-loaded HFF gradually increased in the course of infection and became significant already 1 day after infection at the lowest E:T-ratio of 1:1. Despite the strong gradual increase in inhibition, residual lysis obtained with an E:T of 25:1, however, was still significant even 4 days after infection. Importantly, the observed inhibition was not accompanied by any inhibition of T cell activation, as quantified by degranulation and IFN-γ secretion (**Figures 3B,C**). The levels of secreted IFN-γ thereby were reproducibly markedly higher than obtained with gB-CAR-mediated recognition (**Figure 2B**), likely reflecting enhanced T cell activation, which would explain the observed increased cytotoxicity.

Reduced lysis of HCMV-infected HFF by peptide-specific CTLs is well-known, however, this has been attributed to impaired peptide-presentation and thus reduced TCR signaling (Allart et al., 2003; Manley et al., 2004; Besold et al., 2007, 2009; Kim et al., 2011; Ameres et al., 2014). Our data obtained with the US-gene-deleted HCMV-strain Ad169ΔUS2-11 circumvents this known mechanism experimentally and strongly suggest an additional immune escape mechanism of HCMV.

### Lysis Inhibition Occurs with Different HCMV-Strains and also after Extended Co-culture Periods with the T Cells

To further substantiate our finding, we conducted experiments with the HCMV Towne strain, which, like Ad169, was available with deleted US-gene region. This circumstance allowed the direct comparison of strains Towne and Ad169 both in our CAR-based system and in our experimental EBV peptide specific system dependent on genuine TCR-mediated recognition. Additionally, we tested the possibility that cell death could be efficiently triggered, but progresses with slower kinetics, by prolonging the co-culture-period.

For this set of experiments we again used EBV-BMLF1-specific CTLs and directed them either via their own TCR or by the transiently expressed gB-specific CAR against the HFF. Induction of apoptosis in the HFF (non-infected or 3 days after infection) was determined after co-culture with T cells for 4 and 20 h by flow cytometry. Flow cytometry was chosen, because it allowed us to monitor cells after extended periods of co-culture, which was not possible with the Eu-release assay.

**FIGURE 4:**
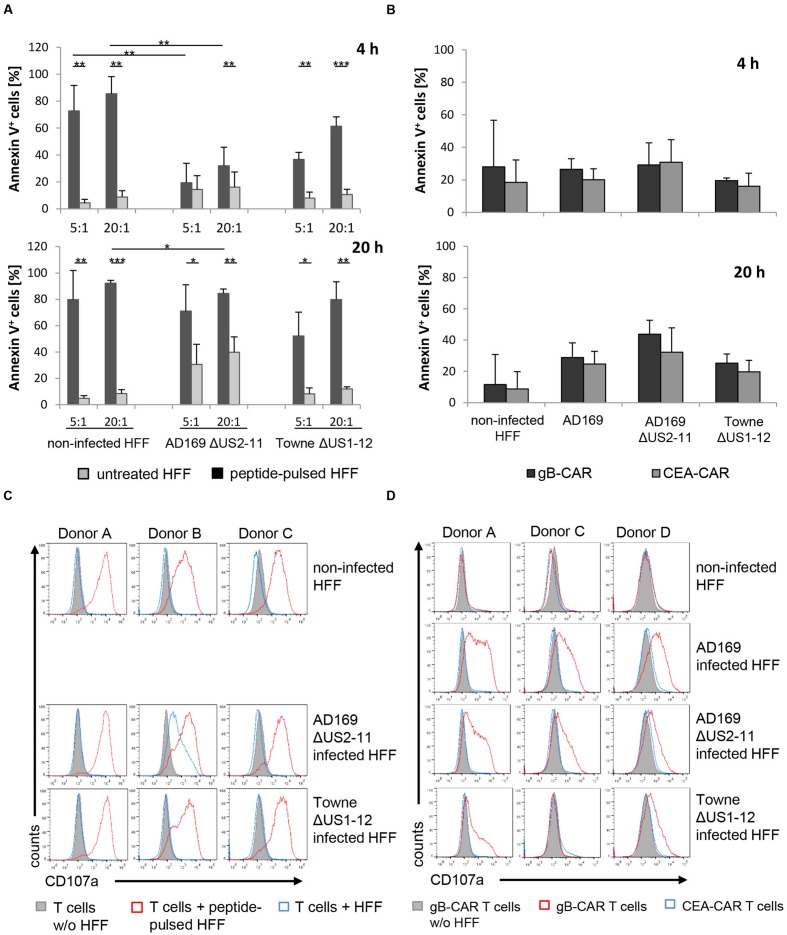
**Lysis inhibition occurs with different HCMV-strains and also after extended co-culture period with the T cells. (A)** Shown is the percentage of apoptotic fibroblasts (non-infected or infected with recombinant HCMV-strains) after 4 and 20 h of co-culture with EBV-specific CTLs (E:T-ratio referring to EBV-specific T cells = 20:1). Apoptotic cells were analyzed by staining with Annexin V by flow cytometry (*n* = 4, 4 different donors). **(B)** Shown is the percentage of apoptotic target cells as indicated after 4 and 20 h of co-culture with CAR-expressing EBV-specific CTLs (*n* = 4, 4 different donors). Contrary to the analogous experimental setup in **(A)**, EBV-specific CTLs equipped with a gB-specific CAR vs. an irrelevant control-CAR were used for comparison instead of target cells plus/minus peptide-loading. **(C,D)** Degranulation of EBV-specific CTLs after co-culture with the indicated target cells, as analyzed by flow cytometric detection of cell surface expression of CD107a. **(A–D)** In all experiments HCMV-infected HFF were used 3 days after infection (MOI 5). **(A,C)** HFF were pulsed with 2 μg/ml of the HLA-A^∗^0201 restricted EBV-BMLF1 peptide GLCTLVAML were indicated.

The lysis of peptide-loaded HFF by EBV-specific CTLs during 4 h of co-culture was significantly inhibited 3 days after infection with Ad169ΔUS2-11 (compared to non-infected HFF, **Figure 4A**), which is in accordance with our previous experiments (**Figure 3A**). Infection of the HFF with TowneΔUS1-12 also resulted in lower levels of lysis by the EBV-CTLs, however, this reduction (compared to non-infected HFF) did not reach statistical significance. When we extended the co-culture period to 20 h, inhibition of lysis of Ad169ΔUS2-11-infected HFF (compared to non-infected HFF) was still present (**Figure 4A**). However, peptide-specific lysis of the HFF (i.e., lysis of peptide pulsed versus non-pulsed HFF) generally increased and became significant for Ad169ΔUS2-11-infected HFF also at the E:T of 5:1.

When we directed, in parallel experiments, the EBV-specific CTLs by transfection of the gB-specific CAR against the infected HFF, we could not find significant CAR-specific cell death induction in any condition, not even after 20 h of co-culture (**Figure 4B**). This is in line with a lower degree of activation of the EBV-CTLs, when they were directed to the infected HFF via the gB-CAR, compared to activation of the EBV-CTLs in response to HFF loaded with saturating amounts of BMLF1-peptide. These differences in activation correspond to the observed different levels of degranulation (**Figures 4C** vs. **4D**); in the case of gB-CAR-directed CTLs this seems to correlate with the levels of gB-expression on the infected HFF (Supplementary Figure S2B).

Taken together, these data indicated that lysis-inhibition is a general function of the Betaherpesvirus prototype HCMV as it is not restricted to a single laboratory strain, and it can also be demonstrated under conditions of prolonged co-culture.

### HCMV-Encoded Proteins Abrogate T Cell Cytotoxicity

The genome of HCMV encodes several anti-apoptotic proteins and untranslated RNAs, which are thought to delay suicide of infected host cells (Brune, 2011). Among them, the proteins UL36 and UL37x1 (vMIA) are known to block death receptor-mediated apoptosis and to inhibit cell death induction at different levels (Goldmacher et al., 1999; Skaletskaya et al., 2001; McCormick et al., 2005). We hypothesized that these proteins might also contribute to inhibition of T cell cytotoxicity.

Skaletskaya et al. (2001) already speculated about a role for UL36, based on its ability to block activation of caspase-8, in the inhibition of T cell cytotoxicity (Skaletskaya et al., 2001). However, the HCMV Ad169 strains used in our study encode an inactive mutant of UL36 (sequencing data not shown) but still mediated resistance to lysis by T cells, indicating that further viral proteins are involved. Furthermore, cytotoxic T cells can induce target cell death independent from caspases by releasing granzymes (Chowdhury and Lieberman, 2008). Nevertheless, we considered it important to directly investigate the ability of UL36 to block T cell cytotoxicity. Furthermore, we also considered the possibility that functional redundancy of multiple factors could prevent loss of function by inactivation of only one factor, as, e.g., UL36. Combined inactivation of these factors in HCMV result in early host cell death (McCormick et al., 2005), and was thus not an option for studying the influence on T cell cytotoxicity in our experiments. Instead, we conducted co-culture experiments of T cells with engineered non-infected HFF that expressed these HCMV-proteins separately and in combination.

**FIGURE 5:**
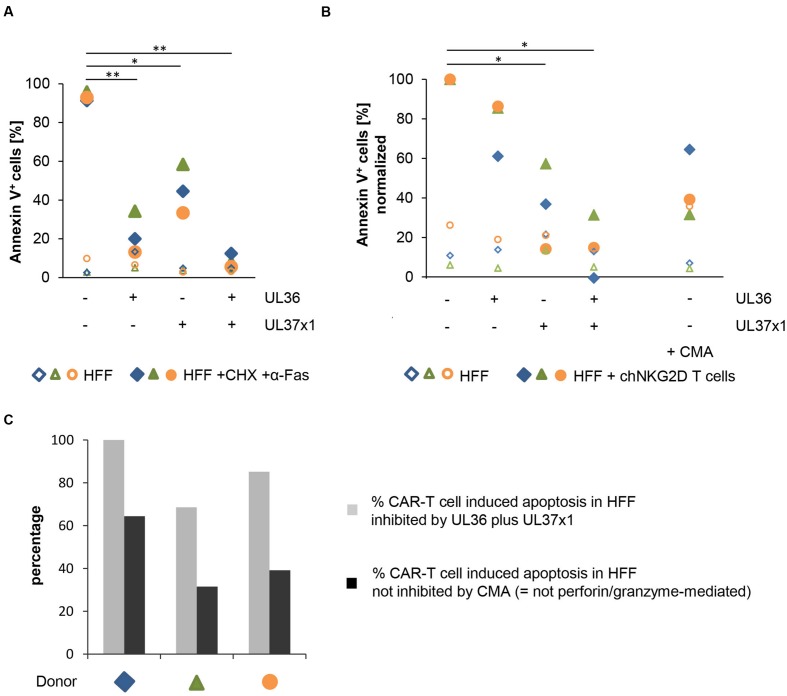
**UL36 and UL37x1 inhibit CAR-T cell-mediated apoptosis in HFF**. The anti-apoptotic HCMV-encoded proteins UL36 and UL37x1 were expressed in HFF by either mRNA electroporation or retroviral transduction, respectively. **(A)** The anti-apoptotic function of the exogenously expressed proteins was confirmed by overnight incubation of the HFF with cycloheximide (CHX, 10 μg/ml) plus an agonistic anti-Fas-antibody (0.2 μg/ml). A GFP encoding vector or mock-electroporation were used as controls. The diagram shows the fraction of apoptotic HFF determined by Annexin V staining in a flow cytometric analysis (*n* = 3). **(B)** The diagram shows the induction of cell death in HFF expressing UL36 and/or UL37x1 after 4 h of co-incubation with chNKG2D-expressing αCD3/αCD28-activated T cells (*n* = 3, 3 different T cell donors). The percentages of apoptotic HFF obtained in the co-cultures of CAR-T cells plus HFF without UL36/UL37x1 expression were set to 100%. Concanamycin A (CMA, 100 nM) was used to block perforin-induced apoptosis. **(C)** The diagram displays the percentage of CAR-T cell-mediated apoptosis in HFF that was inhibited by the combined expression of UL36 and UL37x1 (gray bars; obtained by subtraction of the respective values shown in **(B)** from 100%). The black bars show the proportion of CAR-T cell induced apoptosis in HFF, which was blocked by CMA **(B)** i.e., the cell death that was not mediated by perforin/granzyme. If these values were a result of incomplete CMA blockade, then the black bars (non-perforin/granzyme mediated cell death) would be even lower. The respective differences between the gray and the black bars correspond to the proportion of the perforin/granzyme-mediated cell death in the HFF, which was inhibited by the combined expression of UL36 and UL37x1.

**Figure 5A** shows the results of control experiments that confirm the protective function of the proteins, as previously reported, by blocking death-receptor mediated apoptosis triggered by an agonistic anti-Fas-antibody in combination with CHX (as previously reported by (Goldmacher et al., 1999; Skaletskaya et al., 2001; McCormick et al., 2005). As expected, UL36 strongly inhibited this type of apoptosis; apoptosis was inhibited to a lesser extent also by UL37x1, and most potently by the combination of both proteins.

In order to investigate whether UL36 and/or UL37x1 can inhibit the cytotoxic function of T cells, we used CAR-T cells directed against NKG2D ligands. Experiments with the gB-CAR were not possible, since it was technically not feasible to generate sufficient numbers of non-infected HFFs co-expressing HCMV-gB, UL36, and UL37x1. Since non-infected HFFs in cell culture do express NKG2D-ligands (Supplementary Figure S3B), we could make use of our previously generated NKG2D-based CAR which is highly potent in inducing NKG2D-ligand mediated killing (Lehner et al., 2012). We considered this option straight forward, since the key question of whether and which viral proteins block cytotoxic T cell functions is not a matter of how efficiently those cytotoxic functions are released.

**Figure 5B** shows apoptosis induction in the same non-infected HFFs as used in the control experiments, now targeted by CAR-T cells instead of the Fas agonistic antibody (CAR expression is shown in Supplementary Figure S3A). Indeed, combined expression of UL36 and UL37x1, and to a lesser extent of UL37x1 alone, significantly inhibited CAR-T cell cytotoxicity (*p* = 0.0177 and *p* = 0.0360, respectively). Notably, the potency of either UL36 or UL37x1 to inhibit T cell cytotoxicity was reversed compared to Fas triggered apoptosis (**Figure 5A**), as the inhibitory effect of UL37x1 was strong, and of UL36 was smaller and – in this series of experiments – not significant. As previously analyzed, αCD3/αCD28-activated T cells express perforin and granzymes A, B, K, and M (Supplementary Figure S1E). Blockade of the perforin/granzyme-mediated pathway in the T cells by adding a saturating concentration of CMA, which inhibits acidification of lytic granules and thereby accelerates degradation of perforin (Kataoka et al., 1996), showed a predominant contribution of this pathway (**Figure 5B**). **Figure 5C** compares the extent of UL36/UL37x1-mediated inhibition of HFF apoptosis (gray bars) with the proportion of apoptosis not inhibited by CMA (black bars), i.e., the death-receptor and not perforin/granzyme mediated proportion of cell death triggered by the CAR-T cells. The large differences between the gray and black bars illustrate that within the total of UL36/UL37x1-mediated inhibition of CAR-T cell death (gray bars) there is a substantial proportion of perforin/granzyme-mediated cell death. If CMA-blockade of the perforin/granzyme pathway in these experiments would have been incomplete, then the actual proportions of death receptor-triggered apoptosis (black bars) would even be smaller and the resulting differences (i.e., UL36/UL37x1-inhibited perforin/granzyme-apoptosis) would even be larger.

When we repeated these experiments with EBV-specific CTLs, which were directed via their TCR to peptide-loaded HFF, expression of UL36/UL37x1 in the HFF had a pronounced inhibitory effect with the T cells from one donor, which interestingly secreted the lowest levels of IFN-γ wherease the T cells from the donor with the highest IFN-γ secretion could not be blocked (Supplementary Figures S3D and S3E). Together, the experiments with three donors showed no statistically significant inhibitory effect of UL36/UL37x1. This was not unexpected, since activation of EBV-specific CTLs in response to peptide-loaded HFF possibly triggered maximum cytotoxicity and resulted in significant lysis even 4 days after infection (**Figures 3A** and **4A**), whereas the same EBV-CTLs redirected via the gB-CAR were activated less strongly (reflected in less degranulation; **Figures 4D** vs. **4C**) and could not trigger specific lyis (**Figure 4B**). The fact that HCMV-infection significantly inhibits lysis even under these conditions, whereas expression of UL36 and UL37 in engineered non-infected HFF does not, suggests the existence of yet unidentified further contributing anti-apoptotic factors.

The finding that combined expression of UL36 and UL37x1 in HFF could almost completely block cell death induction by CAR-T cells is in line with our hypothesis that HCMV encoded proteins not only block host cell suicide but also cytotoxic effector functions of T cells.

## Discussion

Targeting of HCMV-infected cells in an HLA-independent manner by CAR expressing T cells is attractive, because it obviates the need for enriching antigen-specific memory T cells and also circumvents one major immune evasion mechanism employed by CMV, which leads to impaired antigen presentation on infected cells by down-regulating HLA class I antigens. Originally, this approach has been proposed for the treatment of HIV using CARs of the first generation and was tested in clinical phase II trials finally (Lam and Bollard, 2013). Recent applications of a CAR T cell approach for fighting Hepatitis B and C have shown promising results both *in vitro* and *in vivo* in a preclinical model (Bohne et al., 2008; Krebs et al., 2013; Sautto et al., 2016). We have analyzed the potential of directing T cells against HCMV-infected fibroblasts by using a CAR recognizing the HCMV gB (Full et al., 2010). However, as reported in this manuscript, in depth studies with this CAR revealed potent anti-apoptotic mechanisms of HCMV, which apparently shield infected cells against cytotoxic effector cell functions.

The Betaherpesvirus prototype HCMV is a complex virus encoding up to 750 translational products in its genome (Stern-Ginossar et al., 2012). Since many of these products are highly immunogenic, HCMV triggers immune responses from all arms of the immune system, resulting in a significant proportion (median ∼10%, up to 40%) of the circulating CD8^pos^ or CD4^pos^ T cell repertoire responding to HCMV-specific antigens T cells (Crough and Khanna, 2009). Despite this strong T cell reaction HCMV persists and establishes lifelong latency, possibly explained by the fact that a large proportion of its genome encodes for RNAs and proteins interfering with the antiviral immune response. One of the most important and intensively studied defense mechanisms of HCMV and its murine homolog MCMV are the prevention of recognition of infected cells by T and NK cells and by antibodies. Like many other viruses, HCMV escapes recognition by T cells through interfering with antigen processing and inhibiting antigen presentation by MHC class I and II molecules on the surface of infected cells. In HCMV this is mediated by the proteins US2, 3, 6, and 11, and possibly by further factors including virus-encoded miRNAs. The activation of NK cells, as a consequence of MHC class I down-regulation and concomitant expression of stress-induced ligands in infected cells, is impeded by down-regulating NK cell activating ligands and by inducing inhibitory signals in the NK cells (Noriega et al., 2012; Halenius et al., 2015). Finally, HCMV is unique by encoding receptors for the Fc domains of all subclasses of human IgG, thereby interfering with IgG-triggered immune responses (Corrales-Aguilar et al., 2014). Together, these mechanisms contribute to interference with recognition of infected cells and the activation of immune effector T- and NK-cells (Rolle et al., 2003; Manley et al., 2004; Tomasec et al., 2005; Besold et al., 2007, 2009; Kim et al., 2011; Prod’homme et al., 2012; Ameres et al., 2013).

We pursued an HLA-independent CAR approach, in order to circumvent immune evasion by down-regulation of MHC I antigen presentation, and also to avoid the need to expand CMV specific T cells not readily available in high risk stem cell transplantation between a seronegative donor and a seropositive recipient. As target antigen we chose HCMV-gB, which is abundantly expressed on the surface of infected cells and which is conserved among different viral strains in the epitope recognized by the CAR. Although our gB-specific CAR mediated effective recognition of gB expressing cells, resulting in efficient lysis of gB transfected 293T target cells and in T cell activation with HVMC infected HFF target cells, neither αCD3/αCD28-activated T cells nor more differentiated EBV-specific CTLs equipped with this CAR could efficiently lyse HCMV-infected HFF. Inhibition of target cell lysis was observed following fibroblast infection with two well-characterized HCMV laboratory strains, i.e., Ad169 and Towne, and also after prolonged co-culture of infected and effector T cells (**Figure 4B**). Further, we showed that this was not a peculiarity of the CAR approach. Inhibition of lysis by HCMV was also evident when an endogenous TCR was involved in T cell triggering, as we demonstrated by using appropriate US-gene-deleted HCMV-strains, EBV-specific T cells and EBV-peptide loaded HCMV infected target cells (**Figures 3B,C**). Together these experiments proved that HCMV blocks HLA-dependent killing by directly inhibiting T cell cytotoxicity. Of note, despite significant lysis inhibition in this peptide dependent experimental system, there was some residual lysis even 4 days after infection (**Figure 3A**), which we had not observed using gB specific CAR T cells (**Figure 1C**). This could indicate suboptimal CAR function, in particular, since also lower levels of secreted IFN-γ and less pronounced degranulation were observed compared to the experiments with peptide mediated TCR stimulation (**Figures 2B** vs. **3C** and **4D** vs. **4C**, respectively). An illustrative example of such suboptimal CAR function resulting in decreased release of effector functions are CARs directed against different epitopes of CD22 (Haso et al., 2013). In interpretation of our data one needs to consider moreover that EBV-specific CTLs were stimulated via their TCR by an unphysiologically high concentration of MHC:peptide complexes on the target cells.

Our data show that HCMV evades the immune response beyond impairing effector cell activation. To our knowledge, only a single study with HCMV-infected astrocytoma cells targeted by HCMV-specific CD4^pos^ T cell clones previously indicated a possible resistance to lysis despite T cell activation, but this was not further investigated (Delmas et al., 2007). On the other hand, while many studies addressed reactivity to peptide-pulsed cells, lysis of HCMV-infected cells has been reported only occasionally. In fact, infected cells can be targeted already early after entry of HCMV through presentation of early expressed proteins such as IE1, and also independently from endogenous viral gene expression through presentation of peptides derived from the ingested viral particles (Riddell et al., 1991; Reddehase, 2002; Bunde et al., 2005; Terrazzini and Kern, 2014). Published findings of efficient lysis of HCMV-infected cells might appear at first sight to be in contrast to our observations reported in this paper, but could be easily explained by differences in experimental setups such as higher E:T-ratios or cytotoxicity assayed very early after infection in these previous reports (Manley et al., 2004; Besold et al., 2009; Ameres et al., 2013).

In search for a possible explanation for the observed resistance to lysis we focused on previously described anti-apoptotic factors encoded by HCMV. HCMV is a particular target of the most ancestral antiviral defense mechanism, namely premature elimination of infected cells by programmed cell death before the viral replication cycle is complete. This is a consequence of HCMVs slow sequentially ordered replication cycle. with immediate-early, early and late phases of gene expression taking 3 days (Mocarski et al., 2013), a process which is paralleled by exponentially increasing levels of viral protein (Reyda et al., 2014), and finally the release of infectious HCMV particles increasing *in vitro* until day 5 after infection (data not shown). In order to prevent termination of viral replication by suicide of its host cells, HCMV has integrated a whole array of repressors blocking the host cell death machinery at several points (Brune, 2011). Anti-apoptotic activity has been described for three HCMV-encoded proteins (UL36, UL37x1, and UL38) and the viral miRNA β2.7 (Brune, 2011). Notably two of them, UL36 and UL37x1, inhibit death-receptor-induced cell death either directly by binding of procaspase 8 in the case of UL36 or, in the case of UL37x1, indirectly by sequestration of the pro-apoptotic protein BAX, thereby preventing BID-induced cytochrome C release from mitochondria and activation of executioner caspases (Goldmacher et al., 1999; Skaletskaya et al., 2001; Arnoult et al., 2004). UL37x1 is further known to inhibit HtrA2/Omi-dependent programmed cell death and might be central for evading host cell suicide (McCormick et al., 2008). The third known protein involved in cell death suppression, UL38, reduces ER stress by so far uncharacterized mechanisms and inhibits the shutdown of the cellular metabolism (Terhune et al., 2007; Qian et al., 2011).

We hypothesized that some of these anti-suicide mechanisms could also inhibit T cell cytotoxicity and we thus investigated the role of UL37x1 and UL36 in this context. Indeed, our data confirmed our hypothesis, because expression of UL36 and UL37x1 in combination and even of UL37x1 alone significantly inhibited the lysis of fibroblasts by CAR-T cells (**Figure 5B**). Our data also show that a major fraction of CAR-T cell cytotoxicity appeared to be mediated by the perforin/granzyme pathway, since addition of CMA strongly inhibited cell death induction (**Figure 5B**; Kataoka et al., 1996). This pathway was also at least partially inhibited by UL36/UL37x1 because the proportion of cell death, which could not be blocked by CMA, i.e., the death-receptor-mediated proportion (black bars in **Figure 5C**), was substantially smaller than the proportion blocked by UL36/UL37x1 (gray bars in **Figure 5C**).

Previously, a role for UL36 in counteracting T cell immunity has been deduced from the ability to inhibit Fas-induced caspase-8 activation (Skaletskaya et al., 2001). In our experiments with cytotoxic T cells, however, UL37x1 appeared more protective than UL36, which was opposite to their relative effects on blocking Fas-induced apoptosis in the control experiments (**Figure 5A** and Supplementary Figure S3C). The small effect of UL36 in blocking T cell cytotoxicity compared to blocking Fas-induced apoptosis might be explained by the large proportion of granzyme-mediated T cell cytotoxicity, which seems to be affected only by UL37x1. This makes it likely that particularly this protein contributes to resistance of HCMV-infected cells to T cell killing. Intriguingly, this matches with the essential role of UL37x1 for host cell survival, which is reflected by the 100% identity of respective protein domains among HCMV-strains (Hayajneh et al., 2001). UL36 seems to have only an ancillary function in both, preventing host cell death and blocking T cell cytotoxicity, since the UL36-deficient Ad169 strain in our experiments showed no increased host cell death and efficiently blocked T cell cytotoxicity. Verification of the hypothesis that UL37x1 has a function beyond preventing host cell suicide by using an HCMV-mutant with inactive UL37x1 was not possible due to extensive host cell death already 3 days after infection before T cell cytotoxicity could be assessed [(Reboredo et al., 2004) and data not shown]. However, further experiments indicated that even multiple factors might additionally contribute to block T cell cytotoxicity, because in our model with strongly activated BMLF1-peptide specific CTLs lysis was not significantly inhibited by the combined expression of UL36/UL37 in non-infected HFF, whereas infection with HCMV strongly inhibited lysis also under these conditions of T cell activation (**Figures 3A** and **4A**). These additional factors could be other anti-apoptotic viral products, but could also include host cell derived factors, such as, e.g., the granzyme B inhibitor SERPINB9/PI-9 (Barrie et al., 2004). The fact that even highly passaged laboratory strains of HCMV have maintained the capacity to strongly inhibit T cell cytotoxicity confirms our hypothesis that the involved factors might at the same time prevent host cell suicide, which is essential also *in vitro*.

In summary, our study shows that the Betaherpesvirus HCMV efficiently shields its infected host cells from cytotoxic attack not only by preventing HLA restricted cell recognition, but additionally also by interfering with a cytotoxic attack through functions of the viral anti-suicide machinery. It might well be that similar mechanisms are effective also with other viruses as many different viral anti-apoptotic proteins have been described so far. In our case it was the use of CAR-T cells, which led to the discovery of this fundamental mechanism and of the ability of UL37x1 to inhibit T cell killing. However, this mechanism does not preclude potential anti-viral efficacy of CAR-T cells. T effector cells also exert non-cytotoxic functions through the secretion of antiviral cytokines and granzymes such as granzyme M, which cleaves cellular and viral proteins important for HCMV replication (van Domselaar et al., 2010, 2013). Thus, HLA-independent targeting of HCMV through CAR engrafted effector cells still seems attractive to pursue, particularly in the case of HCMV-naïve/high risk immunosuppressed patients.

## Author Contributions

Acquisition and analysis of the data (JP, CW, CB, and RG), interpretation of data, and conception/design of the work (JP, ML, AE, FF, and WH), drafting the manuscript (JP, ML, AE, and WH), critical revision and final approval of the manuscript, and agreement to being countable for all aspects of the work (JP, CW, CB, RG, FF, AE, ML, and WH).

## Conflict of Interest Statement

The authors declare that the research was conducted in the absence of any commercial or financial relationships that could be construed as a potential conflict of interest.

## ABBREVIATIONS

CAR: chimeric antigen receptor
CEA: carcinoembryonic antigen
chNKG2D: chimeric NKG2D receptor
CHX: cycloheximide
CMA: Concanamycin
EpCAM: epithelial cell adhesion molecule
Eu: Europium
gB: glycoprotein B
HBV: hepatitis B virus
HCMV: human cytomegalovirus
HCV: hepatitis C virus
HFF: human foreskin fibroblasts
MOI: multiplicity of infection
p.i.: post infection
