# Supplementary material for: Cytomegalovirus-Infected Cells Resist T Cell Mediated Killing in an HLA-Recognition Independent Manner
Source: Front Microbiol. 2016 Jun 9;7:844. doi: 10.3389/fmicb.2016.00844 (PMC4899442; doi:10.3389/fmicb.2016.00844)
Supplement: Supplementary file 1 [file Presentation_1.PDF]

Supplementary Material

Cytomegalovirus-infected cells resist T cell mediated killing independent from HLA-downregulation by expression of viral anti-apoptotic proteins including UL36 and UL37x1

Julia Proff[1,2], Christian Walterskirchen[1], Charlotte Brey[1], Rene Geyeregger[1], Florian Full[3,4], Armin Ensser[3], Manfred Lehner[1*], and Wolfgang Holter[5]

*** Correspondence:** Manfred Lehner: manfred.lehner@ccri.at

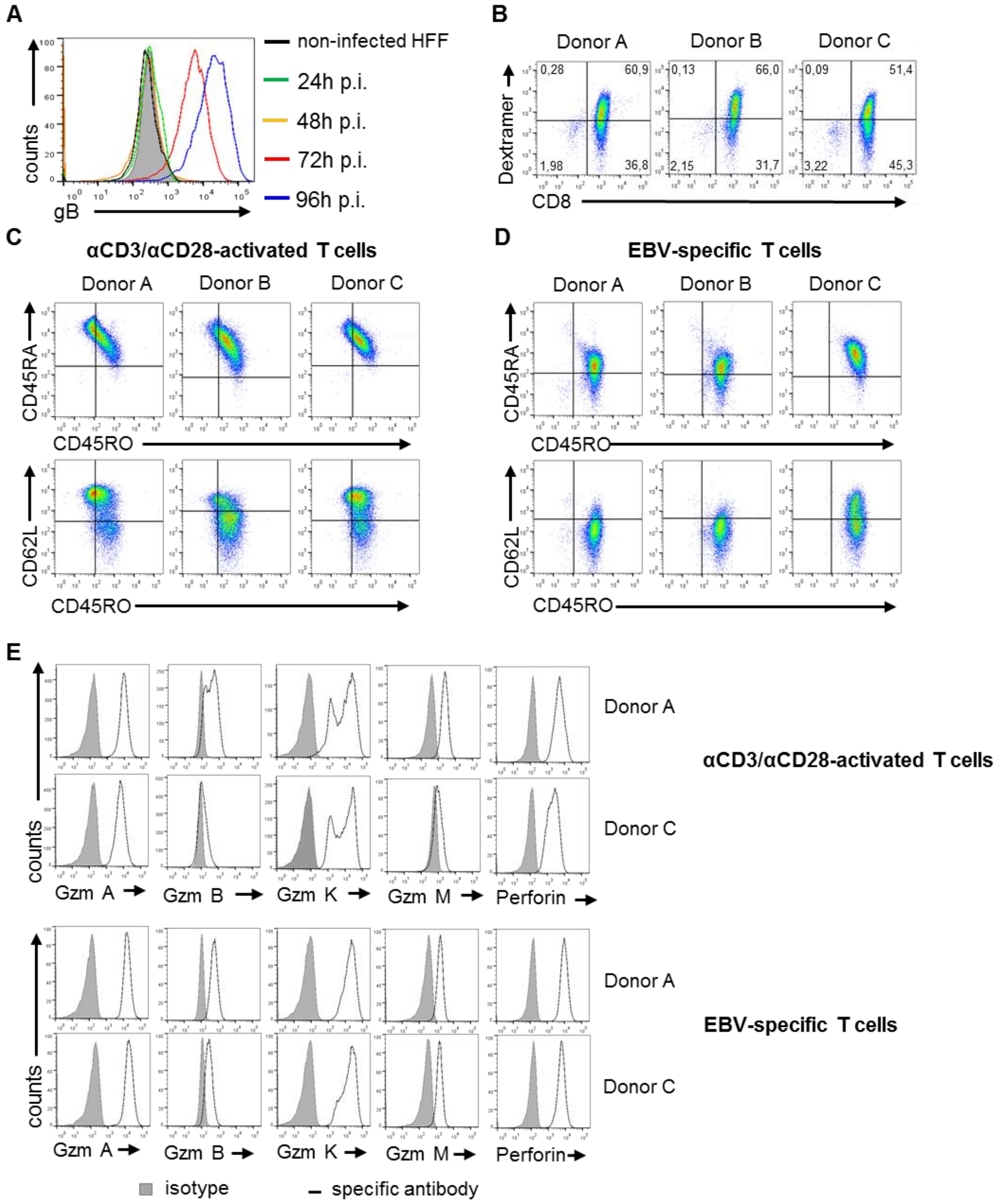

Supplementary Fig. 1: Phenotypic analysis of CD3/αCD28-activated and EBV-specific CTLs. (A) Shown is the flow cytometric analysis of the gB-expression in non-infected and HCMV-infected HFF (AD169, MOI 5) at different time points after infection. **(B)** The frequency of EBV-specific T cells after expansion was analyzed by MHCmultimer staining (Dextramer = HLA-A*0201/GLCTLVAML) by flow cytometric analysis (n=7; 4 different donors). **(C, D)** Flow cytometric analysis of the phenotype of the used αCD3/αCD28-activated and EBV-specific CTLs (non-specific cells included) by extracellular staining of the differentiation markers CD45RA, CD45RO and CD62L (C) and intracellular staining of perforin and granzymes (D).

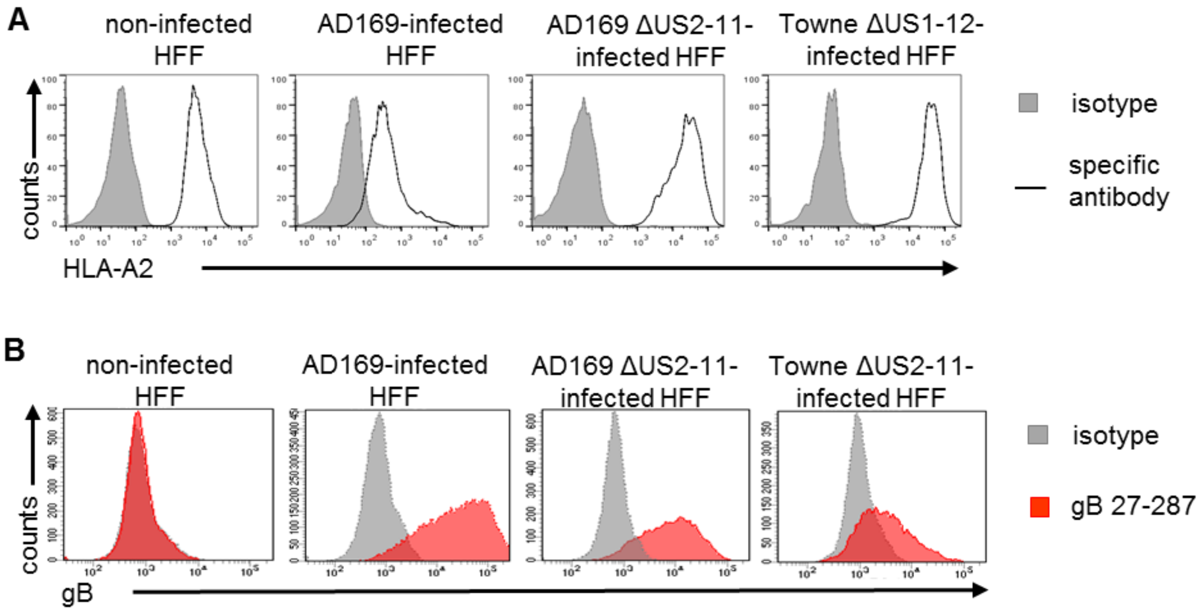

Supplementary Fig. 2: Flow cytometric analysis of HLA-A2 and gB-expression in the used target cells. (A, B) Flow cytometric analysis of the HLA-A2 expression **(A)** and gB expression (using supernatant of the parental hybridoma clone "27-287") **(B)**, respectively, in HFF either non-infected or infected with different HCMV-strains as indicated (MOI 5, d3 p.i.).

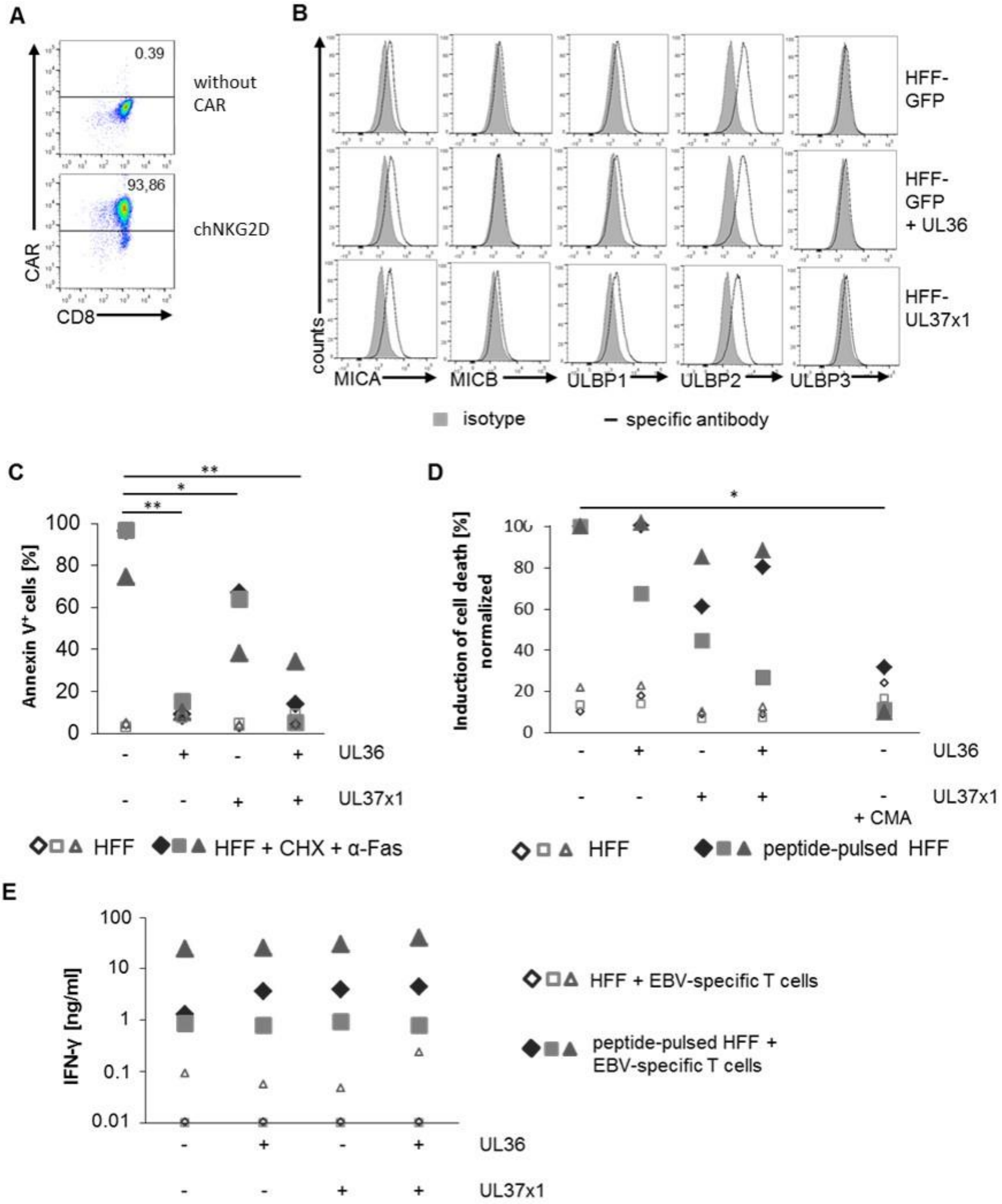

Supplementary Fig. 3: Influence of UL36 and UL37x1 on TCR-mediated cell death induction in HFF. (A) Flow cytometric analysis of chNKG2D expression in αCD3/αCD28-activated T cells 20 h after mRNA electroporation. **(B)** Flow cytometric analysis of the expression of NKG2D-ligands (MICA, MICB, ULBP1-3) in HFF transduced with either a GFP control vector (plus/minus electroporation with mRNA encoding UL36) or a UL37x1 encoding vector as indicated. **(C-E)** The anti-apoptotic HCMV-encoded proteins UL36 and UL37x1 were overexpressed HFF by either mRNA electroporation or retroviral transduction, respectively. **(C)** The anti-apoptotic function of the exogenously expressed proteins was confirmed by overnight incubation of the HFF with cycloheximide (CHX, 10 µg/ml) plus an agonistic anti-Fas-antibody (0.2 µg/ml). A GFP encoding vector or mock-electroporation were used as controls. The diagram shows the fraction of apoptotic HFF determined by Annexin V

staining by flow cytometric analysis (n=3). **(D)** The diagram shows the induction of cell death in peptide-pulsed HFF expressing UL36 and/or UL37x1 after 4 h of co-culture with EBV-specific CTLs (n=3, 3 different T cell donors). The percentages of apoptotic HFF obtained in the co-cultures of T cells plus HFF, which did not express UL36/UL37x1, were set to 100%, respectively. Concanamycin A (CMA, 100 nM) was used to block perforin-induced apoptosis. **(E)** The diagram depicts the levels of IFN-γ released into supernatants upon co-culturing EBV-specific T cells with peptide-pulsed HFF expressing UL36 and/or UL37x1 as indicated. HFF without peptide-loading were used for control (smaller closed symbols).
